# Effect of structural training on surgical outcomes of residents’ first operative laparoscopy: a randomized controlled trial

**DOI:** 10.1007/s00464-018-06657-y

**Published:** 2019-01-17

**Authors:** Ewa Jokinen, Tomi S. Mikkola, Päivi Härkki

**Affiliations:** 1 Obstetrics and Gynecology, University of Helsinki and Helsinki University Hospital, P.O. Box 100, 00029 HUS Helsinki, Finland; 2 Helsinki University Hospital, P.O. Box 100, 00029 HUS Helsinki, Finland

**Keywords:** Surgical education, Resident education, Virtual simulator

## Abstract

**Background:**

Gynecological surgery and resident education have changed during recent decades, thus impacting surgical training. Training on simulators must begin before operating on patients. The objective of this study was to evaluate the effect of a simple curriculum on the surgical outcome of the participants’ first operative laparoscopy.

**Methods:**

This randomized prospective interventional study was carried out in Helsinki University Hospital and Hyvinkää Hospital. We recruited twenty junior residents in Obstetrics and Gynecology, of which half formed a control group and the rest completed the intervention with a theoretical and a practical part. The participants’ first laparoscopic salpingectomy was assessed from video recordings by using Objective Structured Assessment of Technical Skills (OSATS) forms and the Numeric Rating Scale (NRS). The surgical outcome and assessed scores were compared between the groups.

**Results:**

We found no differences in operative time, blood loss, or complications, nor in OSATS or NRS scores. In the intervention group, participants with the weakest performances in the simulator, seemed to benefit from the training program more than the participants with the best performances (skill level elevation 29.2–31.6% vs. 21.1–23.3%, respectively). The participants with the best performances in the simulator were scored among the best in the recorded operations as well.

**Conclusion:**

In this study, we found no difference in the surgical outcome between the groups. However, the participants with low starting levels in the simulator could elevate their skill levels more, though they did not reach the skill level of those with a high starting level. Consequently, we found elevation in skills levels in the simulator tasks, but not in the surgical outcome. Likely, our simple training program with a fixed number of repetitions was insufficient to reach a plateau in the learning curve, and thus the training program in such a curriculum should be proficiency based.

During recent decades, gynecological surgery and training has experienced many radical changes, such as restrictions in working hours [1], new options in medical treatment [2, 3], and delicate office procedures [4]. For residents, this means fewer surgical cases in the operating room.

As live surgical experience is decreased during residents’ surgical rotation [1], they would benefit more from the live cases if they would be well pre-trained. Residents would be able to better focus on pathological anatomy and surgical techniques if they had acquired basic surgical ability and knowledge in a skills lab before operating on patients. More importantly, this would increase patient safety.

Both trainer boxes and virtual simulators have proven to be beneficial in surgical education [5], and now different kinds of curriculums are developed to implement them to training programs [6]. Many published training programs aim to achieve skills to perform a specific operation or task, and they include training with specific procedural modules [7, 8]. In this study, we developed a simple curriculum that contains a cognitive part and a practical part with basic skills only, and evaluated the effect of this curriculum on surgical outcomes of residents’ first laparoscopic salpingectomy.

## Materials and methods

This trial was designed as randomized, interventional, blinded trial with two parallel study groups with 1:1 allocation ration. We recruited 20 residents between June 2013 and December 2016 from the Helsinki University Hospital or from Hyvinkää Hospital. Each new inexperienced resident was invited to participate, and none of them refused. As inclusion criteria, no operative laparoscopies as a primary surgeon were allowed, only diagnostic laparoscopies or clip sterilizations were permitted, as well as to assist in more advanced laparoscopies. Of the participants, ten were randomized to the intervention group, and ten to the control group by a sealed envelope manner. Randomization was done by research assistant outside our study group.

The intervention comprised a web-based theoretical course (Basics in Gynecological Laparoscopy) [9], and of a practical part with LAP Mentor virtual reality simulator (Simbionix Corporation, Cleveland, Ohio, USA). The cognitive material contains knowledge on pelvic anatomy, a laparoscopic technical part including instruments, laparoscopic unit and energy sources, operative phases, gynecological operations and complications, and a test following the actual course. With the simulator, all the nine basic skills tasks were practiced five times each, and the rehearsals were recorded for further evaluation. The nine basic skills tasks included camera manipulating, eye-hand coordination, clip application, clipping and grasping, two-handed maneuvers, cutting, electro surgery, and translocation of objects. From each skill tasks, three to four automatically stored parameters best describing the skill were used in the analysis. The chosen parameters included accuracy rate, total time used, and total path length of an instrument and were 32 in total. Both the web course and the simulator training were required to be completed within a month before the assessment. Both groups took part in similar standard clinical education including patient care in wards and clinical lectures. In the operating room, no operative laparoscopies as a first surgeon were permitted during the study.

The operation to assess the effect of intervention was salpingectomy in the right side; the left tube was alternative if the right one had already been removed. The surgery was video recorded for evaluation. The patients were scheduled to be operated because of a benign condition, commonly salpingo-oophorectomy because of an ovarian cyst or hysterectomy because of abnormal uterine bleeding. In case of salpingo-oophorectomy, first the tube was removed by the resident participating in the study, and thereafter the procedure was continued by the assisting senior. If necessary, the assisting specialist verbally instructed the resident as in any operation where the resident is the first surgeon. Demographics included the resident’s age, gender, working history, numbers of basic laparoscopic operations done, and history of video and instrument playing. The patient data were collected from the medical records and included age, body mass index, previous abdominal surgery, and indication for surgery.

The video recordings were assessed by three assessors (the authors) being blinded for the operator and the randomized group. The assessment was done by using Numeric Rating Scale (NRS) and both OSATS (Objective Structured Assessment of Technical Skills) form for Global Rating Skills (GRS) [10] and salpingectomy-specific form (OSA-LS) [11]. The last item of the OSA-LS form (extraction of the fallopian tube) was dropped off, as in most videos this was not recorded. Before assessing the videos, the assessors made an agreement of concordant use of the forms. Both OSATS and NRS scores were used as the primary outcome measures. The OSATS forms rated score 10–50 (6–30 from the GRS and 4–20 from the OSA-LS) and NRS 0–10 using expert level as a reference. The secondary outcome measures included operating time, blood loss, and direct complications. Data for operating time and direct complications were collected later in the medical records, while blood loss was visually graded in the recorded videos using stages 0 to 3.

Power calculations were based on OSATS scores. According to previous study [12], in laparoscopic salpingectomy, the difference in OSATS scores between novices and intermediate group was six points. Aiming to detect this difference and using type 1 error 0.05 and power of 0.80, the study required at least 18 participants and thus, we included 20 participants, 10 in each group.

The statistics were done by using SPSS 21.0 statistical software (Chicago, IL). The analyses were done by the first author (E.J.) with guidance by our statistician. The reliability of the three assessors was calculated by Intraclass correlation coefficient using both absolute and consistency type agreement. To investigate differences between the intervention and the control group, for continuous variables, we used the Independent samples T-test for parametric and Mann–Whitney U Test for non-parametric variables. For categorical variables, we used Pearson Chi-Square test.

The study plan was approved by the Helsinki University Hospital Ethics Committee (Dnro390/13/03/03/2012) and from the Hospital District of Helsinki and Uusimaa.

## Results

**Fig. 1:**
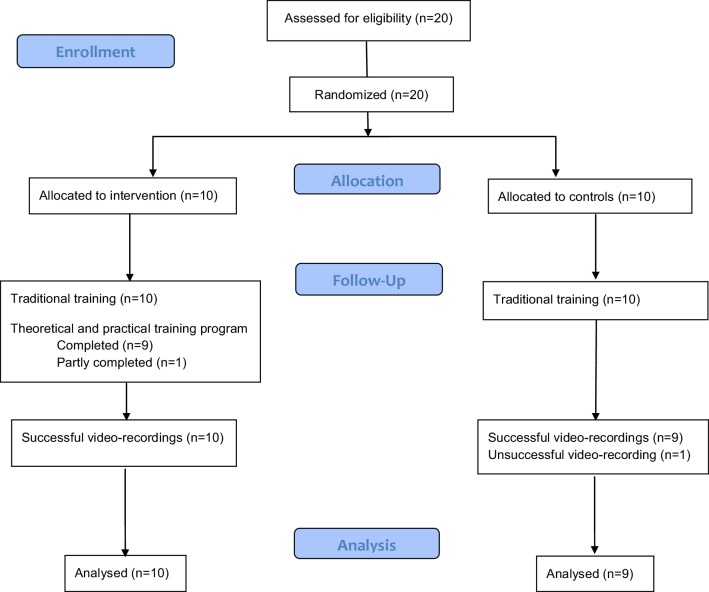
Flowchart of the participants

Flowchart is shown in Fig. 1. One of the video recordings failed in the control group, and this participant was excluded from the study. One participant in the intervention group did not complete the training program due to unknown reason, but the recorded operation was included in the analysis.

Patient, operator/participant, and surgery-related demographics are shown in Table 1. There were no differences between background data concerning operated patients. Participants in the control group had longer experience in general surgery, but not in gynecology, and there was no statistical difference in basic laparoscopies done. Otherwise there were no differences between the groups.

**Table 1:** Patient, operator/participant, and surgery-related demographics

	Intervention group mean (standard deviation)	Control group mean (standard deviation)	p-value
Patient related			
Age (years)	52.8 (13.6)	53.6 (10.1)	0.893
BMI (kg/m²)	25.1 (4.2)	24.1 (4.2)	0.608
Previous abdominal surgery (n)	0.7 (0.5)	1.0 (1.0)	0.428
Deliveries (n)	1.4 (1.1)	1.3 (1.1)	0.996
Operator related			
Age (years)	32.9 (3.0)	32.2 (4.0)	0.711
Experience in Obst & Gyn (months)	17.5 (4.7)	18.1 (12.5)	0.898
Experience in General Surgery (months)	3.0 (3.2)	5.9 (2.4)	**0.034**
Basic laparoscopies done (n)	0.1 (0.4)	1.2 (1.7)	0.120
Video playing (no/yes/ex)	6/1/1^a^	7/0/0^b^	0.364
Instrument playing (no/yes/ex)	4/1/3^a^	6/1/0^b^	0.187
Surgery related			
Operative time (min)	14.6 (4.7)	12.6 (4.0)	0.349
Bleeding (stage 0–3)	0.8 (1.0)	0.3 (0.7)	0.515
Operated side (right/left)	8/2	6/3	0.628
Complications (n)	0	0	

Statistically significant values are highlighted in bold (*p* < 0.05)

^a^Data of 8 participants

^b^Data of 7 participants

**Fig. 2:**
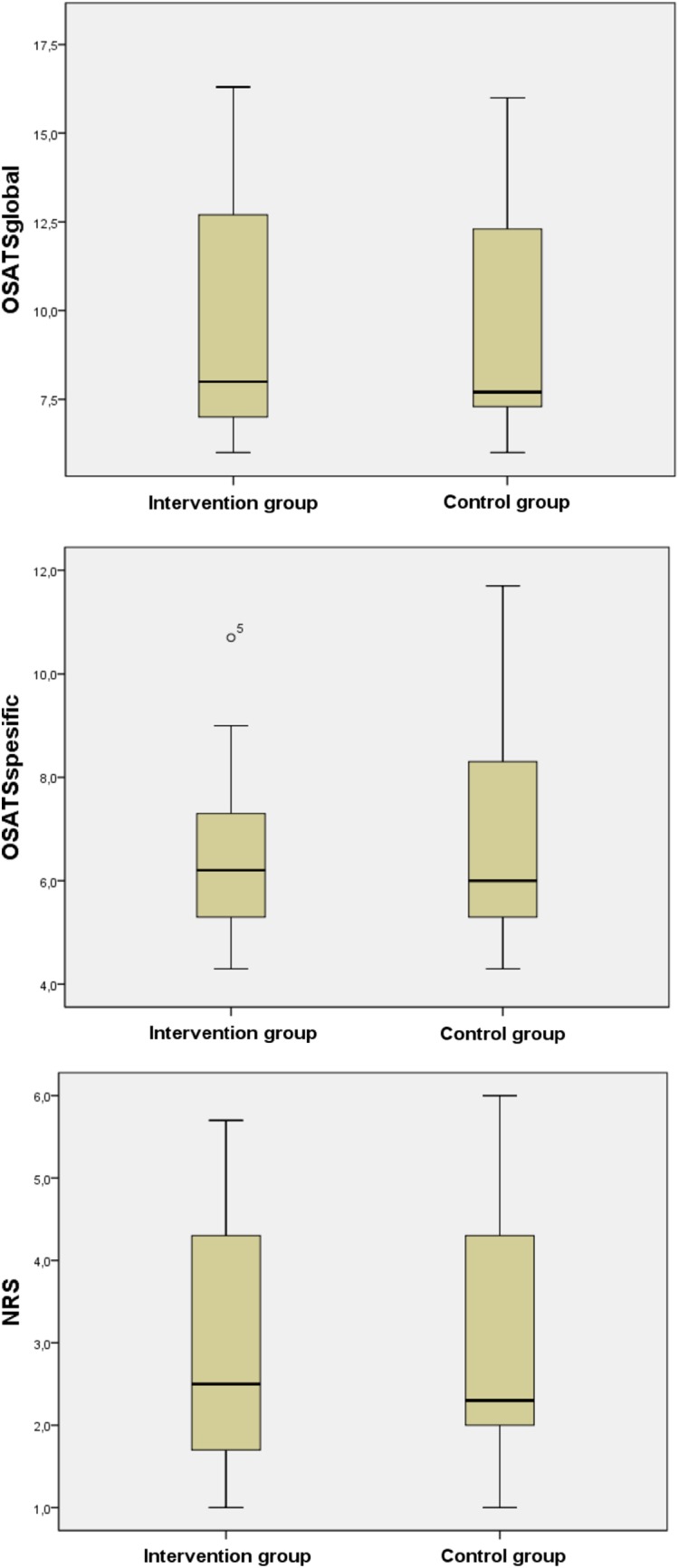
OSATS points and NRS scores in the study groups. Line represents the median value, box 50% of the cases, and whiskers the whole range

In assessing the recorded operations, there were no statistical difference in OSATS or NRS scores between the control and intervention group (Fig. 2). The mean for total OSATS points was 16.4 (SD 5.3) in the intervention group and 16.2 (SD 5.7) in the control group, and the mean NRS was 2.9 (SD 1.6) and 3.1 (SD 1.7), respectively. In the reliability analysis, between the three assessors, the consistency type agreement was 0.787 (95% CI 0.729–0.835) and the absolute type agreement 0.787 (95% CI 0,729-0.834). In the operations recorded, there were no differences between the groups in operative time, bleeding, or complications (Table 1).

**Fig. 3:**
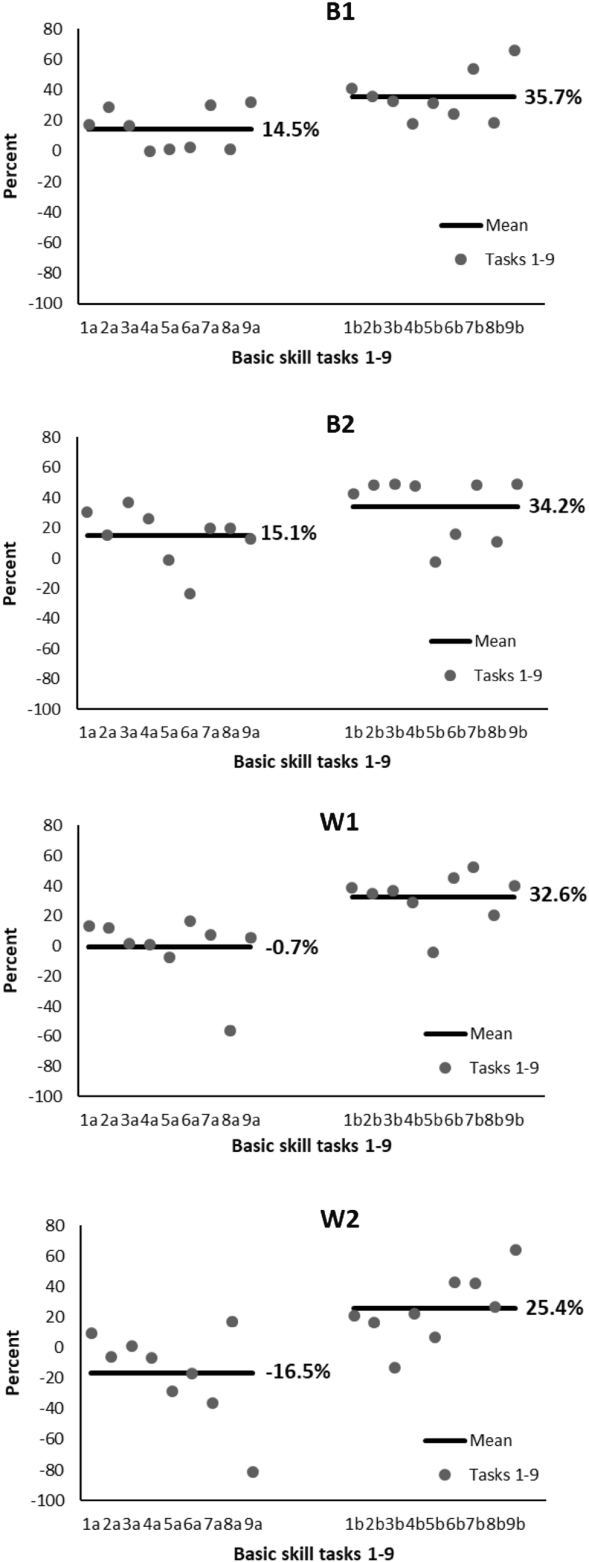
Skill levels in the beginning (**a**) and in the end (**b**) of the training program in each task. Results of only two of the best (B1 and B2) and two of the weakest (W1 and W2) are shown. The level is shown as difference in percent using the study group mean in the beginning of the program as a reference

**Fig. 4:**
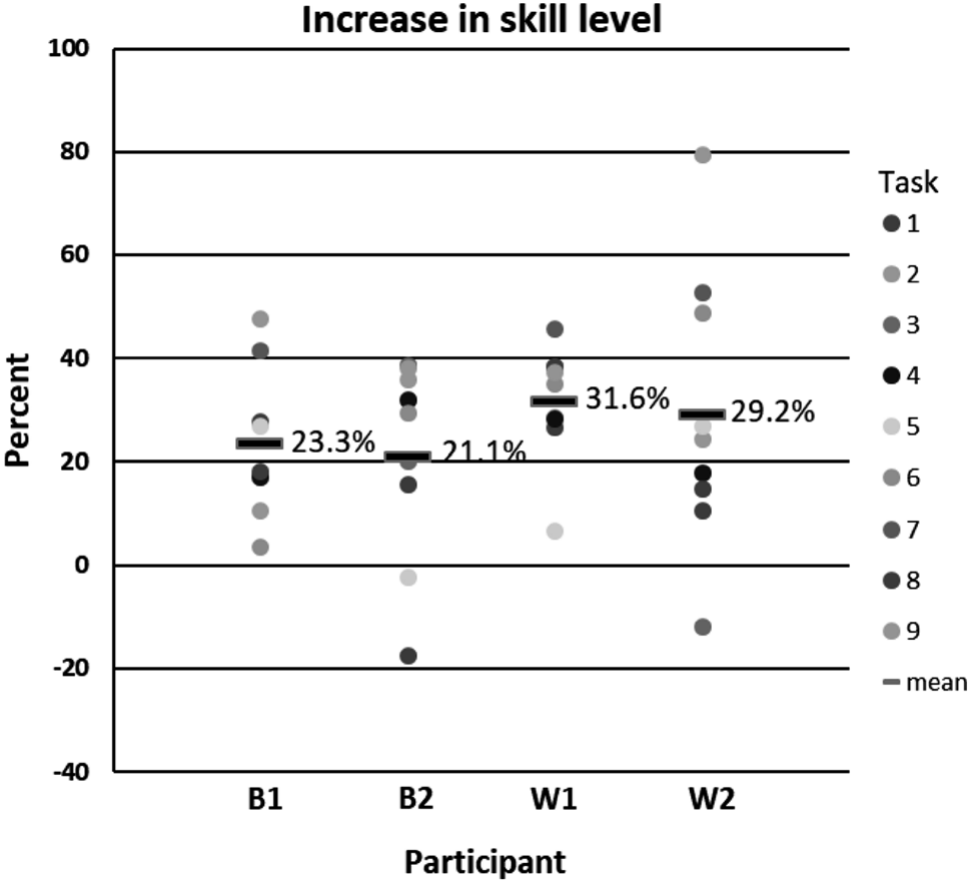
Increase in skill level in percent during the training program in each task. Results of only two of the best (B1 and 2) and two of the weakest (W1 and 2) are shown

The data recorded in the simulator were visually analyzed for defining the plateaus in the learning curves by using group means in each skill task and in the chosen parameters. Plateau was reached in two of the nine skill tasks; in cutting and in translocating of objects. In recorded data, by visually comparing individuals’ performances in the chosen 32 parameters, participants whose performances were two of the best and two of the weakest were clearly distinguishable. We evaluated their practice program thoroughly. When comparing the skill level in the beginning of the practice program, the best performed participants started 14.5% and 15.1% above and the weakest performed participants 0.7% and 16.5% below the whole group mean (Fig. 3). We found a tendency that the best performed participants elevated their skill levels 23.3% and 21.1%, while the skill levels were elevated 31.6% and 29.2% among the weakest performed participants (Fig. 4). At the end of the training program, the skill levels of the best performed participants were still higher (35.7% and 34.2%) than those of the weakest performed ones (32.6% and 25.4%) (Fig. 3). When comparing the performance in simulator with the assessment scores in the real operation among intervention group, the best participants in simulator were scored 2nd and 3rd, while the weakest performed were scored 9th and 10th .

## Discussion

In our randomized study, we found no difference in the surgical outcome between intervention and control group. As an intervention, we used a combination of a theoretical web course and a training program with a virtual simulator.

In our intervention, the training program in a virtual simulator comprised of basic skills tasks only, and their repetition of five times each. We noted a broad distribution in starting levels and learning curves. In overall data, only in two tasks the learning curve plateau was reached, indicating that the intervention was insufficient. The starting levels and learning curves have been noticed to be highly variable between individuals [13, 14], and in different studies, the repetition rate to reach the expert level was broad [8, 15, 16]. In a study investigating the effect of training basic skills modules in a LapMentor virtual simulator, 10–22 repetitions in different tasks were required to reach expert criterions [17]. However, there is no fixed expert criterion, although the target level for training affects training length, skills level assessment, and training costs [18], but the expert criterion level is often determined by the experts in their own clinic. Programs with proficiency-based interventions improve OSATS scores in salpingectomy [12, 14] and in tubal occlusion [19], but operating performance was improved also with fixed training time in tubal occlusion [20, 21] and in salpingectomy [22], and with fixed training session numbers in cholecystectomy [23]. In a recent study, junior residents’ skills improved after repeating LapMentor basic skills modules five times in a single session [24]. Despite these different findings, proficiency-based training programs seem to be more reasonable than programs based on fixed repetitions or specific training time.

Interestingly, when evaluating skill levels in the simulator data, we noted that residents with low starting levels improved the performance more than residents with higher starting levels. However, at the end of the training sessions, their skill level did not reach the level of the residents with a higher starting point. According to our study, there seems to be a tendency between virtual simulator skill levels and operative skills, as the best performing participants in a simulator also had the best OSATS scores in operations, and vice versa. Both findings are in concordance with an earlier study according to which training in a virtual simulator is most valuable in the early stages of training and a better score in a virtual simulator is predictive of better surgical performance [25].

We included only basic skill tasks in our intervention, as our goal was to investigate preparedness for real operations, and we chose to use a virtual simulator. Some curricula contain a technical skills training program with basic skills in a low-fidelity model (FLS, ESGE), while others have a training program with virtual simulator containing either basic drills [23] or both basic drills and procedural modules [8]. Basic skills training has been proven to turn to better procedural skills in the operating room using in training both low- [14, 20] or high-fidelity [20, 23] models. In addition to technical drills or procedural modules, a laparoscopic curriculum should contain theoretical knowledge [26, 27]. We included a web course ‘Basics in Gynecological Laparoscopy’ [9] available for every resident in Finland through the internet as a cognitive part. As a consequence, the whole program became self-guided and easy to be trained. The contents of our theoretical course are relatively concordant to cognitive components in laparoscopic curricula published in England [6], North America [28], and Denmark [8]. Some curricula have the theoretical knowledge as a self-learning material on a separate CD-ROM, like Fundamentals of Laparoscopic Surgery (FLS) program [29] from the Society of American Gastrointestinal Endoscopic Surgeons, or on-line, like the Winners program from the European Society for Gynaecological Endoscopy (http://www.esge.com). Some curricula have lectures on similar subjects [8, 22], or have both self-learning and lectures [30].

One of the strengths of our study is assessing live operations, as the main object in resident training is better performance in the operating room and increased patient safety. Also recordings of the operations were all successful, assessors were blinded for the operator and the study group, and reliability between assessors was good. In a review of virtual simulation education in laparoscopy [31], from 9 published studies using a live human operation for assessment, only one was in gynecology. After this review was published, a live tubal occlusion was assessed after virtual reality training in two studies [19, 20]. In addition, a live salpingectomy was assessed after training basic skills with a box trainer [14] and after diverse intervention including salpingectomy in a porcine cadaver [22]. Thus, simulator training studies assessing live operations are still rather few and therefore, further studies are still needed.

Our study has limitations. First, recruitment of participants took more than three years. Fortunately, recruitment bias was avoided, as every suitable resident agreed to participate. During that time, there were no changes in traditional teaching. Nine of ten participants in the intervention group completed the rehearsal program with the simulator as instructed. All training sessions were documented and assessed. Second, the sample size is small, and thus, chance could have had an impact on the study outcome. In the control group, there were three participants whose performance without training reached the best performances in the intervention group. As expected, the basic psychomotorical skills differs between individuals. Since the study group was rather small, these three talented individuals may have affected the result to the extent that possible differences between the study groups could not be detected.

To conclude, with our simple systematic training program, we were unable to show a significant effect on residents’ first laparoscopic operation. During training sessions, the plateau in the learning curve was not reached in every task, thus a proficiency-based curriculum could be more effective. It appears that residents with lower baseline laparoscopic skills benefit most from training with a virtual simulator.
